# Breaking the cycle of panic and neglect: better preparedness for the next pandemic

**DOI:** 10.1016/j.lanwpc.2025.101586

**Published:** 2025-05-30

**Authors:** The Lancet Regional Health – Western Pacific

5 years after the first COVID-19 case, WHO member states have reached a consensus to strengthen future pandemic prevention, preparedness, and response. We have experienced multiple cycles of panic and neglect in outbreaks: ramping up efforts during outbreaks and then withdrawing resources once the immediate threat subsides. Will this time be different?

The Asia–Pacific region has been the epicentre of outbreaks such as severe acute respiratory syndrome, avian influenza, and recent COVID-19, and remains at high risk of disease outbreaks due to multiple factors like diverse wildlife, high population density and mobility, frequent human–wildlife interactions, climate change, and rapid urbanisation. The region also bears a disproportionate burden of infectious diseases like malaria, tuberculosis, antimicrobial resistance, and other re-emerging infections.

Given the intensity and scale of environmental, demographic, and socioeconomic changes and interactions, another pandemic in the future is inevitable. The key question is how well prepared we will be. Pandemic preparedness requires a country to have national response plans, resources, and the capacity to react to pandemic outbreaks. While many countries in the Asia–Pacific region have established pandemic preparedness plans and enhanced infrastructure and capacity building after multiple health crises, the response to prevent the recurrence of another health crisis is far from sufficient. The lack of sustainable financial investment in pandemic preparedness—shaped by political will and available resources amid post-COVID-19 economic recession and political uncertainties—is one of the key reasons for insufficient preparedness. The Global Burden of Disease 2021 Health Financing Collaborator Network suggested that funding for pandemic preparedness is available, but what is needed is to prioritise these resources through strong political commitment. However, it is hard to justify investment in tackling health threats that have not happened. Besides, achieving effective public health preparedness requires coordinated management and funding sources, which are usually lacking in many low-income and middle-income countries that are most affected by infectious diseases.

We cannot afford another pandemic, considering both short-term and long-term health and economic loss in a health crisis. There is a need to change the mindset; health spending on disease surveillance and diagnostic capacities is not a cost but an investment that will benefit infection control and enhance the resilience of health-care systems. The early success in COVID-19 control in many Asia–Pacific countries has shown that investment in health-care and public-health infrastructure capable of effectively managing the next outbreak is worthwhile. As people in the most vulnerable situations are most affected by pandemics, it is moral to have coordinated and sustainable financing mechanisms from domestic and international partners for pandemic prevention and preparedness. While the benefits of reducing pandemic risks are global, implementation primarily takes place at national and subregional levels. Considering each country varies in its level of pandemic preparedness, there is a need to set up a framework defining health priorities and core investments for future preparedness. In the long term, we need to study how local and global investments have been translated into capacity-building and pandemic preparedness, and what capacities have been sustained and integrated into health-care systems after COVID-19.

The emergence and evolution of pathogens highlight the urgent need for continual innovation, as seen with the rapid development of mRNA and viral vector vaccines for COVID-19. Years of investment have positioned many countries in the Asia–Pacific region as leaders in science and technology. Rapid advances in artificial intelligence, multi-omics, and telehealth have transformed infectious disease surveillance, diagnosis, and health-care delivery. Countries in the region have evolved from being vaccine producers to innovators, developing more effective and accessible vaccines such as intranasal and thermostable formulations. Locally developed innovative solutions will enhance the Asia–Pacific region's preparedness for pandemics and infectious disease outbreaks. With well-established vaccine manufacturing capacity, diagnostic platforms, and digital tools, regional innovations can be scaled up to support global health efforts.

Many challenges remain in the world after the COVID-19 pandemic: long COVID persists with no remedies; pathogens with outbreak potential are evolving, but we don't know what, when and where the next will be; and health inequities are increasing with no systematic mechanism to resolve them. This time, we must break the cycle of panic and neglect by emphasising pandemic preparedness investment and health innovation to find out why certain health interventions fail to prevent disease outbreaks, and to better prepare for the next pandemic.

